# Retention of Upper Dentures

**Published:** 1914-12-15

**Authors:** Loomis P. Haskell


### RETENTION OF UPPER DENTURES.

BY LOOMIS P. HASKELL, D.D.S.

In the discussion of the subject of the retention of upper dentures, perhaps the experience of seventy years, devoted exclusively to plate work, might throw some light on the subject, at least to the young men coming into practice in great numbers every year.

For thirteen years previous to the introduction of vulcanite, metal work prevailed exclusively.

My preceptor, Dr. M. P. Hanson, practicing in Salem, Mass., and later in Boston, was one of the first—in fact I think, was the first—about 1843, to use plaster for impressions and to make a suction plate, and I will relate the occasion.

Using the common plaster in vogue for models, he secured a satisfactory impression. For the die he used tin. As lead cannot be poured upon tin, it was necessary to cast the counterdie first. This was done by drying the model and holding it in the lead until it was hard; then, knocking out the plaster, a rim of several thicknesses of paper was placed over the counterdie, and the tin was poured. The gold plate was then swaged. Upon trying it in the mouth the adhesion was so strong that he wished to test it, which he did by soldering to the center of the plate a loop to which was attached a long wire holding suspended a common water-pail filled with water; this the patient lifted from the floor before the plate became detached. Previous to that time dentures had been narrow plates held in place by spiral springs carried inside the cheek; from that time, however, my preceptor had no further use for springs. The plate came into close contact with the membrane all over the surface, and the result was adhesion. Later on, Levi Gilbert, a dentist of New Haven, introduced the air-chamber, which soon came into general use. I used it for many years, but of late years have seldom found occasion for its employment.

The theory of the retention of upper dentures is simple. Every dentist knows that the center of the palate is hard, but does not seem to consider that it is the only portion of the jaw that does not change, whereas the alveolar border does change—under vulcanite, on account of its non-conductivity, changing excessively in seventy-five per cent. of cases; under the metal plate also changing to some extent. Unless provision be made for such change it is only a question of time when the plate, instead of resting on the ridge, will be resting hard on the palate and rocking.

To overcome this difficulty, in making a metal plate I place on the model a "relief," employing the usual base-plate wax, extending from near the top of the ridge and covering the entire hard surface, averaging in width half an inch, somewhat narrower at the anterior, and thinning at the margin so as to leave no distinct impression. The plate must of course extend at least an eighth of an inch beyond the relief. In a vast majority of cases plates can be worn farther back than as usually made.

A test of this principle is found in the heavy continuous gum denture in flat jaws. In these instances I never use an air-chamber. And if it is not needed in these cases, where is it needed? And if weight does not count here, where does it count?

There is a small percentage of cases (about 3 per cent.) in which the palate is soft, and usually in these instances a crevice is observed. In such cases I employ neither relief nor air-chamber, but simply fit the plate close to the membrane. In vulcanite dentures, scrape the impression or the plate when it is being finished.

There are more failures from faulty occlusion than from any other cause. The anterior teeth must never come in contact, the pressure coming on the bicuspids and first molars.—*Dental Cosmos.*

